# How parents choose to use CAM: a systematic review of theoretical models

**DOI:** 10.1186/1472-6882-9-9

**Published:** 2009-04-22

**Authors:** Ava Lorenc, Yael Ilan-Clarke, Nicola Robinson, Mitch Blair

**Affiliations:** 1 Centre for Complementary Healthcare and Integrated Medicine, Thames Valley University, Paragon House, Boston Manor Road, Brentford, Middlesex, TW8 9GA, UK; 2 Lifespan Research Group, Royal Holloway, University of London, 11 Bedford Square, London, WC1B 3RF, UK; 3 River Island Paediatric and Child Health Academic Centre, Imperial College, Northwick Park Hospital Campus, Watford Road, Harrow, Middlesex HA1 3UJ, UK

## Abstract

**Background:**

Complementary and Alternative Medicine (CAM) is widely used throughout the UK and the Western world. CAM is commonly used for children and the decision-making process to use CAM is affected by numerous factors. Most research on CAM use lacks a theoretical framework and is largely based on bivariate statistics. The aim of this review was to identify a conceptual model which could be used to explain the decision-making process in parental choice of CAM.

**Methods:**

A systematic search of the literature was carried out. A two-stage selection process with predetermined inclusion/exclusion criteria identified studies using a theoretical framework depicting the interaction of psychological factors involved in the CAM decision process. Papers were critically appraised and findings summarised.

**Results:**

Twenty two studies using a theoretical model to predict CAM use were included in the final review; only one examined child use. Seven different models were identified. The most commonly used and successful model was Andersen's Sociobehavioural Model (SBM). Two papers proposed modifications to the SBM for CAM use. Six qualitative studies developed their own model.

**Conclusion:**

The SBM modified for CAM use, which incorporates both psychological and pragmatic determinants, was identified as the best conceptual model of CAM use. This model provides a valuable framework for future research, and could be used to explain child CAM use. An understanding of the decision making process is crucial in promoting shared decision making between healthcare practitioners and parents and could inform service delivery, guidance and policy.

## Background

There is considerable debate around the definition of Complementary and Alternative Medicine (CAM) [1-3], definitions varying over time[4]. CAM can be defined as "any health improving technique outside of the mainstream of conventional medicine"[2]. One of the most recent definitions divides CAM into mind-body medicine, biologically based therapies, manipulative and body-based systems and energy medicine, and whole system approaches such as Ayurveda and Traditional Chinese Medicine[5].

CAM is very popular, with recent population based estimates of yearly adult use in the UK of 20% – 28%[6,7] and 34% – 38% in the USA [8,9]. A systematic review of CAM prevalence surveys worldwide found a prevalence of between 23% – 62%, and for over the counter CAM 25% – 46%[10]. CAM is also commonly used for children, with prevalence estimates from 12% in the USA[9], 11% in Canada[11] to 51% in Australia[12]and 17.9%[13] to 37%[14] in the UK. There is evidence that home remedies are also commonly used for children in the UK[13,15,16].

As in making choices about conventional care treatments, in choosing CAM there are numerous considerations to take into account. Such considerations revolve around the personal perception of the balance between expected drawbacks of action (e.g. side effects) and the anticipated benefits of the treatment[17]. Within this vast continuum, the variables which will impact on the decision making process include desires (utilities associated with each alternative, personal values, goals, etc), beliefs (expectations about processes and outcome, knowledge, means to achieve desired outcome etc)[18] and other practical considerations (e.g. access). Decision making in healthcare may be moving from paternalism to autonomy, finally settling on shared decision making and 'consumerism'[19].

In addition, the decision making process for child health is different to that of adults as it may include the whole family not just the patient[20]. Decisions are often made by parents, not the child although there is debate around the role of children, with the model of constrained parental autonomy suggesting that parents make decisions for their children but this autonomy is not absolute[21]. Family centred care is now a central tenet of healthcare, particularly nursing[22], although a recent literature review found that despite the importance of including children in decision making on their own health they are rarely involved in the decision making process[23]. Regarding CAM use, particularly for certain ethnic minority groups, children may be even less autonomous than in other areas[24,25]. Adolescents however are likely to have a greater degree of autonomy, using CAM due to personal beliefs and control[26] Parents may use CAM to be a 'good' parent, particularly for children with a serious illness[27]. In addition parents may not use the same treatments for children as for themselves, including home remedies[28]. Parents may be more cautious with children's health, trusting and visiting practitioners more readily and taking caution in using home remedies[25]. However, they may also feel more strongly dissatisfied with conventional healthcare for their child than for themselves[29]. In addition, females are higher users of healthcare service[30], and mothers are more likely to use CAM for their children than fathers[31,32], indicating that mainly mothers are involved in decision making.

There is a large amount of literature written on the decision-making process in conventional care but the extrapolation of the conventional medicine (CM) decision-making process to CAM choices is debatable[33]. The process of choosing to use CAM may be far more dynamic, iterative and more individualistic than the more logical and rational decisions in conventional care[33,34].

The literature about choosing to use CAM encompasses many different approaches. Many studies have identified the factors associated with CAM use in children in the UK[13,15,35,36], the USA[32,37-42] and Canada[11,43]. However, much of the research into reasons for using CAM so far has been atheoretical and lacks a clear and comprehensive conceptual framework to contain and explain the processes which are inherent in CAM decision-making[44,45]. Most of the studies are based on survey methods, are cross-sectional and cannot determine directional relationships[46]. In recent years there has been a move to explain the mechanisms which motivate and actualise the choice of CAM.

This review examines the literature relating to the decision-making process in CAM, but excludes models which do not include psychosocial factors or affective values or beliefs such as computational models of decision making, the cognitive processes involved in decision making (e.g. Hypothetico-deductive model[47]) and other descriptive theories of the decision making process which relate to treatment choices (e.g. Prospect theory[48]). This review focuses on the decision which leads to choosing CAM; as such it concerns itself with the models which attempt to explain the choice of complementary or alternative healthcare and the psychosocial factors that are involved in this decision.

Two dominant approaches have been used to study decision-making in CAM: the first originates in the concept of healthcare utilisation, concerned with the factors which enable and encourage the consumption of health services. The second approach views the decision to use CAM as a health behaviour, where the decision to use CAM is viewed within the framework of social and psychological, mainly cognitive, factors. Background on the models reviewed is given in table 1.

**Table 1:** Descriptions of the models

Model	Description	References
Andersen's Sociobehavioural Model (SBM)	Sets out three sequential components which mitigate healthcare use. The first most indirect, are predisposing factors including beliefs, sociodemographics and characteristics which motivate the healthcare service use. The next component, more directly related to behaviour, are enabling factors which allow and give access to healthcare services (e.g. income, physical location, insurance). The final most proximal component is medical need, including the objective and subjective experience of symptoms of illness	[65]
Consumer Decision-Making model (CDM)	Has three components: first is external influences: sociocultural influences on beliefs, knowledge and behaviours. Second is the consumer decision-making process; including psychological influences (values, beliefs, attitudes, personality) which form the main part of the decision making process. Finally, the post decision behaviour consists of the behaviour itself and an evaluative comparison of the actual experience with the anticipated experience.	[51]
Health locus of control (HLoC)	The HLoC identifies the extent to which people perceive their health, treatment, course of illness and other health related factors, to be under their control or external to them (e.g. fate, doctor, others).	[55,61]
Transtheoretical model (TTM)	TTM engenders five stages: 1. Precontemplation (no intention to make change) 2. Contemplation (consideration of making change) 3. Preparation (effecting small steps to begin change) 4. Action (carrying out the change to its full extent) 5. Maintenance (sustaining the change over time). The distinguishing characteristics of this model are firstly that moving through the stages is not necessarily a linear process, but it is necessary to move through all changes in order to incur sustained change; Secondly, the balance of pros and cons of carrying out a given behaviour, will determine the stage of change in which the individual finds him/her self.	[92]
Theory of planned behaviour (TPB)	TPB attempts to explain behavioural intentions as predicted from three major sources: attitudes, perceived behavioural control and subjective norms. Attitudes include beliefs and expectations about a particular behaviour and the extent to which consequences are seen as desirable. Subjective norms are the beliefs one has about the expectations of 'significant others' and the motivation to comply with these. Perceived behavioural control is the extent to which one expects the behaviour to be easy or difficult and whether they perceive themselves to have the ability to carry out such a behaviour – often equated with self efficacy	[57].
The self-regulatory model (SRM)	The SRM explains how individuals have 'illness beliefs' or 'illness perceptions' about their condition. These are predefined cognitions which represent illness characteristics and coping strategies, related to perceived cause, effects, consequences, duration and sources of control or cure. People go on to form a representation of their coping alternatives, which may be represented as 'treatment beliefs'.	[70,93]
Braden's Self-help model	Braden's self-help model specifies central variables and relationships involved in a learned response to chronic illness and includes the following elements: side-effects burden, uncertainty, perceived enabling skills, self help and quality of life. Its utility lies in its ability to form the connection between individual's use of enabling skills to manage their illness.	[72]

### Healthcare utilisation models

The decision leading to choice of healthcare can be modelled by pathway (sequential), or determinants models[49]. Pathway models give stages of healthcare seeking, moving from self-care, adoption of the sick role, seeking medical care and finally recovery[49]. Determinants models focus on explanatory factors of the choices made[49].

The main determinants model is Andersen's sociobehavioural model (SBM)[30,50], particularly prominent in the (conventional) medicine literature[30]. This model sets out three sequential components which mitigate healthcare use; predisposing, enabling and need factors[30]. A recent addition to the model is the role of social support, whereby social influence can encourage the utilisation of healthcare as well as the perception of the efficacy of a given treatment[50].

The Consumer Decision-Making model which is less often used for healthcare has three components: external influences, the consumer decision-making process and the post decision behaviour[51].

The majority of healthcare utilisation models have found that the dominant determinant of healthcare utilisation is need, or illness[49].

### Health behaviour models

Health behaviour models take into account psychological influences on behaviour and thus explain the individual differences in behaviour, but often do not take account of external characteristics such as sociodemographic variables. Within this approach there are various models which have been applied to health behaviours, such as the Health locus of Control (HLoC), the Theory of Planned Behaviour (TPB), the Transtheoretical model (Stages of change) (TTM) and the Self-Regulatory model (SRM).

The concept of HLoC originates in Attribution Theory [52] and relates to the extent to which individuals view events as under their control (an internal locus of control) or out of their control (external locus of control). HLoC has been widely applied in the health and other arenas [53,54], based on the prediction that individuals high in internal locus of control are more likely to carry out health-promoting behaviours, whereas those with high external locus of control who attribute their health to chance will be unlikely to engage in health-enhancing activities [55]. The TTM was originally developed as a charting of the processes engendered in the elicitation and maintenance of change during the therapeutic process [56] and has been used for various health behaviours. The theory of planned behaviour[57] attempts to explain behavioural intentions as predicted from three major sources: attitudes, perceived behavioural control and subjective norms. The SRM was formulated to describe the process whereby when confronted with a health threat, individuals seek overcome the problem and return to normality.

This paper presents the results of a systematic review to identify how these models have been applied to the prediction of CAM use, and focuses on their potential use for children. The paper discusses the methods used in the literature search, followed by the results which discuss the quality of the papers found and their conclusions regarding the suitability of the model they test.

## Methods

### Aim

To identify a conceptual framework which can successfully model the parental decision making process of choosing to use CAM for children, through a systematic review of studies using a decision making model for CAM use.

A systematic search was conducted of the following databases: Psychinfo, Sciencedirect, Academic search elite, Medline, Psycharticles, Elsevier, Biomed, Ingenta connect, Cinahl and Embase. A combination of the following search terms was used: CAM or Complementary or alternative Medicine, choice, decision making, parent or child or adolescent or paediatric or pediatric, model, utiliz*.

**Figure 1:**
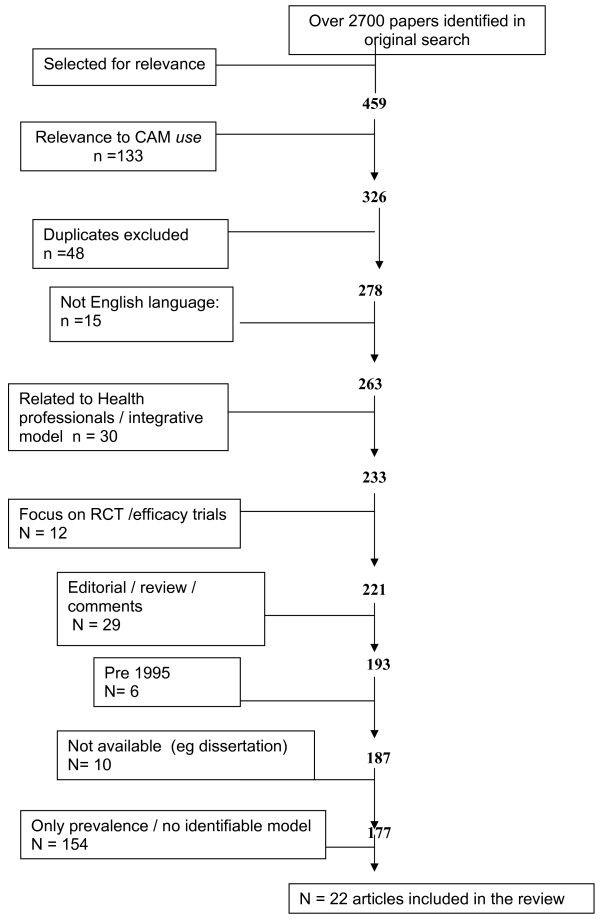
**Flowchart of article selection process**.

Two stages of screening were used to identify relevant articles. A diagram of the process of inclusion/exclusion is given in Figure 1.

At first screening exclusion criteria were: randomised control trial/efficacy trials of CAM; physician/practitioner knowledge/choice/decision making; teaching in medical school; integration into primary care; non-English language; relating to regulation of CAM; survey, comment, editorial or review; published before the year 1995.

At the second selection stage the inclusion criteria were that the paper had to present a *model *of factors associated with the utilisation or choice of CAM. This was done by screening each paper for the key word 'model' or 'theory' and determining their use or reference to a model within the article (excluding the term 'regression model' and 'integrative model/integrative care model'). The term model was occasionally used in reference to a *paradigm*, rather than the intended use as in a theoretical framework which depicts a process of sorts; when this occurred it was necessary to exclude such studies.

## Results

Over 2700 articles were screened for inclusion. Only one study focussed on children, so the review had to include articles related to adult decision making. As seen in Figure 1, 22 articles met the criteria for inclusion in the review. These studies are presented in Additional file 1, and discussed below.

### Models

In the current review 7 established models and four 'unidentified' models were used to predict the psychological process of choice of CAM: scales relating to the locus of control [58-62], TPB [63], TTM [63], Andersen's sociobehavioural model [29,58,64-69], the self-regulatory model [70,71], the consumer decision making model [44] and Braden's self-help model [72]. Six studies [71,73-77] constructed models based on their findings, including one using ethnographic decision tree modelling[71]. Only one study focussed on child use of CAM[29].

### Design

Out of 22 studies surveyed, 16 used quantitative approaches to examine the models in question. Six studies [71,73-77] used qualitative methodology. In the initial search, many studies were identified which used qualitative methodology to examine the subject of predictors or correlates of CAM use, however in the subsequent analysis these had to be excluded due to lack of use of a model.

All the studies, excluding one [67] used a cross-sectional design. The main disadvantage of such a design is that cause and effect cannot be determined in spite of collection of a large amount of variables. This often resulted in largely correlational datasets, which, while being informative and to some extent predictive, fail to provide a causal model of CAM decision-making and to identify the exact mechanisms through which CAM choices are made. Only one study examined the propensity for CAM use over two time points, thereby assigning specific causal relationships to use of CAM[67].

One major advantage of a number of studies was that they used the frequency of CAM use as a variable to compare specific predictors of more or less use. By doing this they were able to distinguish between the beliefs which differentiated a person who tried CAM but did not have a committed treatment plan [58].

### Sampling

The majority (10) of studies surveyed had medium sized samples of between 123 and 551, indicating attempts to achieve a representative population. Of the studies using self-selection there was a predominance of female respondents, usually because females were more likely to use health services [78] and more likely to use or consider using CAM [6,8,79-81]. Some studies were specifically aimed at the experience of females only, mainly in relation to breast cancer and other female-dominated diseases [61,64,72,73,75], one included males only (prostate cancer)[76]. Other studies based on large scale, often national, studies had much larger samples of between 1672 and 31,044 so were able to pursue a more representative sample in most cases. Qualitative studies had much smaller samples, between 16 and 42, which is appropriate for their methodology.

Overall, the response rate for most studies was highly acceptable at 60% or higher for most studies[82]. This serves to enhance the reliability of the findings in terms of their generalisability. However, for a few studies there was very low response rate, which would indicate that it was unlikely that the sample was representative of the population concerned[44,60,62].

Most studies only included adults, most defined as over 18, but some with limited age ranges[65,70,71,76]. Only one paper [29] focused on the use of CAM for children and utilized a theoretical model.

### Settings

Studies were mainly conducted in the US, Canada, Japan and the UK (although limited by English language inclusion only). Participants were recruited from conventional medicine (CM) centres, CAM clinics, health related internet sites, national surveys or random internet mailing.

### Measures and analyses

Most of the quantitative studies included in this review tended to use measures which were largely found to be reliable and valid. This was often established in previous studies which used the same measures. In some cases, the reliability of the measures was tested through internal consistency (Cronbach ά) and multi-item responses used to establish reliability within the studies. Aside from the qualitative studies and a number of the surveys, many of the studies were self-report questionnaires, which are open to biases in the form of response bias, demand characteristics and to the introduction of systematic errors.

The qualitative studies used semi structured or open ended interviews. They also tended to use methods which attest to the integrity and validity of the data such as confirming the findings with participants. One qualitative study tested the predictability of the model they developed[71].

The quantitative studies predominately used multivariate logistic regression or multinomial logit regression to explain the relative variance of each of the factors significant in the decision making process although some analyses were limited to bivariate or correlational association[59,66]. The qualitative studies used either grounded theory[73,75], or 'thematic' analysis[74,76,77].

### Limitations of the studies

Many studies did not distinguish between different types of CAM, which may have significant implications given that those studies that did differentiate CAM type found that the decision making process did vary for the different CAM modalities[66,68-70]. Some studies were unclear about what was included in their definition of CAM[67,72].

Not all studies controlled for factors which may have biased the sample or introduced extraneous variables, such as; stage of illness, duration of illness and conventional or other treatments[61,67,72]. Studies using the SBM did not always explain the recursive nature of the factors which has recently been described[50].

Although some studies were based on large, nationally representative samples, some used small, potentially underpowered samples[58,59,62]. Two studies additionally only included CAM users, preventing comparison of CAM users and non users[58,75]. Although most studies did not specifically exclude non CAM users, there may have been response bias in terms of CAM users being more likely to take part.

A number of studies used non validated measures, which limited the validity of the study and also makes comparison between different studies difficult. In particular a number of the studies based on the SBM used non validated measures of health beliefs [65,66], or did not include health beliefs at all[68,69]. Some studies did not provide statistics on the percentage of variance the model explained[59,66].

## Discussion

The current review found that almost 100 papers (eliminated from the final analysis) did not use an overarching framework to examine their findings, leaving them open to spurious explanations. Some studies investigated psychological constructs such as beliefs, using validated measures, but refrained from going further to consolidate a model. Other studies set out to validate the items and their interrelationships within a model, discussed below (see table 1 for descriptions of models).

### CAM definition

In the studies included in this review not all studies made the distinction between different types of CAM therapies. Hendrickson et al highlight the problem of treating CAM as one modality and illustrate through their study that there are differences in the determinants of use of different type of CAM therapies[68]. Most studies included only practitioner based therapies, others viewed CAM in terms of the behaviour. The lack of a consistent operational definition of CAM use made the papers heterogeneous and difficult to combine and form conclusions and may explain some of the inconsistent findings in the literature. There is a need to examine studies which identify types of CAM in order to compare their findings, reflected in Andersen's suggestion that the outcome of the SBM should ideally relate to a specific type of healthcare service[50]. In addition, a distinction between CAM use as a treat, a preventative strategy or treatment of disease was often lacking; This may serve to distinguish between diverse CAM users who have different motivations for using such services[66] In addition other factors influencing the status of CAM which may affect the decision making process will vary between, and even within, countries of study. These include professional regulation, legal status, financial access and reimbursement of CAM and its integration within national health systems.

### Healthcare utilisation models

The socio-behavioural model was the most commonly used, and was largely supported for modelling CAM decision making, although some studies were only partially supportive of the model; most commonly enabling factors were not significant. One study[69] was based on an adapted SBM model, the "CAM Healthcare Model"[83]. Here the SBM was extended to examine the concurrent, complementary use of conventional medicine and CAM, and the choice between them, and included self-care practices and products as well as practitioner based CAM[83]. The only study using the SBM for child CAM use added the component of healthcare experience[29]. These adaptations may be important for child use of CAM which is often non practitioner based (88% of CAM use by London paediatric outpatients[84] and 64% in the USA was non practitioner based[85]), and parental use of CAM is very likely to influence child use[11,13,29,40,86,87].

**Figure 2:**
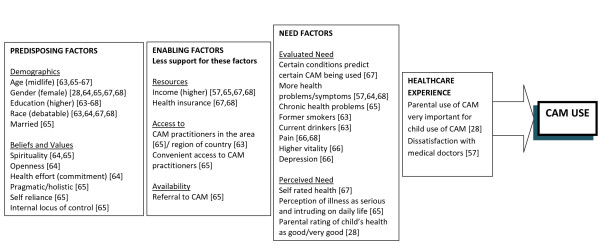
**Factors in the SBM important for CAM use**. References show which factors were important in which studies.

The findings from the studies using the SBM are summarised in Figure 2. As described by Andersen[50], the importance of health beliefs and organisational (enabling) factors may be underestimated due to the inadequate conceptualisation (and therefore measurement) of these in many studies. Findings have supported Andersen's claim that need factors are important, but it should be emphasised that these factors are heavily dependent on social context and health beliefs[50].

This review only identified one study using a theoretical model for child use of CAM. Although this study found support for most components of the SBM, this study was based in the USA, only tested practitioner based CAM, and the survey was not specifically designed to test the SBM. The lack of studies on child use of CAM using a theoretical model means this review is unable to extrapolate findings to the use of CAM in children. However it does highlight the need to identify the most suitable model and to test it's suitability for application to CAM use both for parents and children to determine whether the processes are similar to the choices of CAM in adults. The original SBM used the family as the unit of study[30], and although recently this has changed to the individual, due to problems in measuring family based variables[50], the family focus is especially appropriate for child CAM use.

One of the main strengths of the SBM is that it incorporates variables which may include both subjective (e.g. health beliefs, perception of illness) and objective (e.g. income, symptoms) variables from a variety of domains – socioeconomic, biological, psychological and social. As such this model fits very comfortably with an interdisciplinary and integrative view of healthcare utilisation, whist taking account of idiosyncratic influences as well.

However, the SBM falls short of naming the specific processes which are often complicated and non-linear, which lead to the specific decisions to use CAM among individuals and subgroups[44]. Also, qualitative studies [73,77] highlighted the importance of temporal factors, e.g. deciding to use CAM through continuous appraisal of well-being and due to perception of circumstances at that point, which SBM fails to account for. While the model succeeds in incorporating health beliefs within the predisposing factors, which in turn encompass other affective and cognitive factors, specific health beliefs are often not identified. Empirical study using the SBM needs to ensure that the findings are integrated back into the model and not left as a collection of associations. To this end, it is recommended that future studies utilise a longitudinal prospective approach, whilst ensuring differentiation between different CAM modalities.

The Consumer Decision-Making model[51] was able to take into account the variability in the decision factors and the intricacy of their relationships and effects on each other. However, it is not specifically related to health and does not include factors relating to emotional and interactive aspects of care which may be important for both CAM use and child healthcare. The Consumer Decision Making model contained no integration of affective or value-laden factors which would differentiate individuals with similar experience from one another[44].

### Health behaviour models

When CAM use is viewed as a health behaviour, individual differences which are not explainable in terms of more extrinsic characteristics (e.g. socio-demographic) can be explained. This is important given that CAM use is often a behaviour specific to an individual[77]. The advantages of this approach are two-fold; Firstly psychological factors, such as cognition, beliefs and values, are considered to be important and proximal to the decision to carry out certain behaviour, and may mediate other more extrinsic factors[88]. Secondly, psychological factors, as opposed to extrinsic factors, have the advantage of being amenable to change, at least to some extent; this is particularly important in relation to health-related interventions including CAM[88].

The health locus of control was predictive of CAM use in two studies[59,61] but not in three[58,60,62]. In addition, many of the HLoC studies were carried out on samples of patients with a chronic illness, so of limited generalisability. In terms of the decision to use CAM, it can relate to the perception of control over illness and treatment.

TTM and TPB were found to be supportive of CAM use prediction and psychological factors were more important than medical or demographic, but only when the two models were used together, and findings may be open to selection bias[63]. Both beliefs about the positive effects and worries about the negative effects were important[63]. Family expectation was particularly important in the TPB[63].

The self-regulatory model received little support for predicting CAM use[70]. People may pursue a particular treatment if they perceive their illness in a certain way or hold particular treatment beliefs, for example having a holistic approach to health and illness being the strongest predictor of CAM use[89]. Braden's self help model had strong support as patients used CAM because it was perceived as effective and was related to income, however it was only tested by one study of cancer patients[72].

The Health Locus of Control, Self-Regulatory model, TPB and TTM all had the weakness that they tended to originate from a singular viewpoint which results in limited integration of the sources of influence, and thus they were able to account for limited variability in the dependent variable.

The models developed using qualitative data may prove useful once empirically tested, although these were all based on patients with chronic health problems[71,75,77].

### Limitations of review

Due to disparate terms used in the decision making literature, it was difficult to define search terms to capture all relevant papers. Although searches were kept broad with extensive hand searching of reference lists to capture a wide range of articles, the search terms could have included terms such as 'Framework', although subsequent scanning of results it did not seem to make a difference to the papers chosen for inclusion. Language bias may well be an issue as only English language papers were chosen (15 were excluded for this reason) [90].

The review only included published papers, which did not capture potentially important sources of information such as theses, conferences abstracts and official reports. In addition papers on this subject were published in journals in a very wide range of subject areas; there may be other databases which should have been included. The review only included studies published post 1994 which may have been a source of bias.

Terms for CAM could have been expanded to include all CAM modalities (e.g. acupuncture, herbal etc), in order to capture studies that may not be indexed under general CAM terms.

### Future research

The review found that the SBM has strong support for modelling the decision making process in CAM use. However, a number of methodological limitations were identified which future research needs to address. The decision making process appears to vary depending on the CAM modality; comparison between CAM modalities should be made. Extraneous variables should be controlled for, especially illness characteristics. Quantitative studies should include sufficiently powered samples, validated measures and multivariate analysis. Studies of the SBM should also incorporate the dynamic, interactive nature of the factors in the model.

The use of qualitative methods to explore decision making is particularly interesting and should be considered carefully. The discipline of psychology is particularly prone to quantitative methods, except when the subject of study is exploratory. This issue may represent a disadvantage in applying psychological approaches to the data as many pertinent findings which arise from qualitative studies are often omitted from subject analysis as they do not fit easily into pre-set conceptual categories. As child use of CAM was identified as an underexplored area, the use of qualitative methodology to examine the predictors and correlates of CAM use would be particularly relevant for child use of CAM.

Some of the authors are now engaged in a funded research project using qualitative methods to clarify validity and to identify the relative importance of the factors and will test this using a quantitative questionnaire using correlational and regression analysis to validate the model.

## Conclusion

Andersen's sociobehavioural model has been identified as a suitable model for modelling the decision making process resulting in adult CAM use. However, the suitability of application of this model to child CAM use has not fully been studied and needs further clarification. This identification of a suitable decision making model is facilitating theory-guided research into how and why CAM is used for children, through empirical testing. Using an existing model promotes methodological consistency, which is imperative in the field of CAM which often uses disparate methods and tools. Providing an overarching model which has been tested for a child population will aid to guide healthcare practitioners' understanding and application to clinical practice[91].

## Abbreviations

CAM: Complementary and alternative medicine; CM: Conventional medicine; SBM: Sociobehavioural model; HLoC: Health Locus of Control; TTM: Transtheoretical model; TPB: Theory of planned behaviour; SRM: Self regulatory model.

## Competing interests

The authors declare that they have no competing interests.

## Authors' contributions

MB conceived of the paper. NR supervised the project. YIC carried out the literature searches and YIC and AL reviewed individual papers. The manuscript was drafted by AL and YIC and revised and amended by MB and NR. All authors read and approved the final manuscript.

## Pre-publication history

The pre-publication history for this paper can be accessed here:



## Supplementary Material

Additional file 1 **Results of literature review**. This table gives detailed information on each study included in the review, including study design, factors predicting CAM use, limitations and suitability of the model tested. Click here for file
