# Supplementary material for: How parents choose to use CAM: a systematic review of theoretical models
Source: BMC Complement Altern Med. 2009 Apr 22;9:9. doi: 10.1186/1472-6882-9-9 (PMC2680396; doi:10.1186/1472-6882-9-9)
Supplement: Additional file 1 — Results of literature review. This table gives detailed information on each study included in the review, including study design, factors predicting CAM use, limitations and suitability of the model tested. [file 1472-6882-9-9-S1.doc]

| **Additional file 1: Results of literature review** | | | | | | | | | | | | |
| --- | --- | --- | --- | --- | --- | --- | --- | --- | --- | --- | --- | --- |
| **Name, year, country of study** | **Population, setting, sample, age range** | | **Design and statistical analysis** | **Measures (and their validity)** | **Factors predicting CAM use** | | | | | **Limitations of study** | **Suitability of model for CAM use** | |
| **ANDERSEN’S SOCIOBEHAVIOURAL MODEL** | | | | | **Predisposing** | | **Enabling** | **Need** | |  |  | |
| Hendrickson et al [67]  2006  USA | Stratified sampling – to ensure representativeness  (60% response) n=2166  Adults (no age range given) | | Descriptive, cross- sectional telephone survey.  Logistic regression analysis | - Predisposing: age, gender, education, race, residence,  - Enabling: Income, health insurance  - Need: Self-rated health, experience /frequency of 11 health problems,  - Outcome: Dichotomous measure of CAM use (differentiation between different types of CAM) | - Biologically based CAM:  age, gender, education.  - Mind-body therapies: age, gender, education.  - Manipulative CAM: Gender, race, residence.  - Whole CAM: Race | | - Biologically based CAM: health insurance.  - Mind-body therapies:  income.  - Manipulative CAM: income  - Whole CAM: income, insurance, | - Biologically based CAM: self rated health, back pain, insomnia, allergies and anxiety.  - Mind-body therapies: self rated health, arthritis, allergies, digestive problems + anxiety.  - Manipulative CAM: arthritis, back pain, allergies.  - Whole CAM:  self-rated health, back pain, insomnia, allergies, anxiety | | No health beliefs / attitudes examined.  Sample not entirely representative of other states – not varied – mostly white, high income+ health insurance | **Supportive of HBM use (although beliefs not measured).**  **Variation in different factors (all three components) according to type of CAM.**  **No social**  **Influence** | |
| Upchurch, et al [63]  2008  USA | N = 17,112 (100% female)  (74.3% response rate).  Adults (women) 18+ yrs | | Cross sectional, in person household interview.  Bivariate stats and logistic regression.  Part of a large scale survey (NHIS) | - Predisposing factors: Race, education, nativity, age, marital status  - Enabling factors: Income, employment, health insurance  - Medical need and personal health practices: Self-reported health status, smoking, alcohol, BMI,  - Outcome: use of acupuncture | Race, education, age.  (association with race dependent on educational attainment) | Region of the country. | | Former smokers and current heavy drinker | | Acupuncture only.  Did not supply info on % of variance which model explains.  Excluded those who classed themselves as ‘other’ in terms of their race. | **Study supportive of utility of SBM in predicting CAM use**  **Factors from all the 3 domains of SBM and personal health practices predictive.** | |
| Hildreth et al [70]  2007  USA | Nationally representative Response rate for whole survey (60%). In total this study n=1672  Adults 31-64 yrs | | Cross sectional telephone interview and follow up mail questionnaire.  dataset from Life in the US study MIDUS  Negative binomial (linear) regression. | - Predisposing factors: demographics/socioeconomic and social support, non validated single items about health beliefs such as religious world view, (n=4) health control, health effort and openness  - Enabling factors: socioeconomic  - Need: no of health conditions and functioning  - Outcome: Conventional healthcare use + any CAM use. | Female, white, educated. Higher spirituality, openness Health effort (commitment) | None | | More health problems, except when functional disability. | | Non validated health beliefs questions. | **Only predisposing and need factors, (not enabling) predicted CAM use** | |
| Kelner & Wellman[65]  1997  Canada | N=300: 240 CAM users (75% female), 60 CM users (68% female). From healthcare practices.  Adults 18+ yrs | | Cross sectional. Face to face semi-structured interviews (quantitative and qualitative analysis).  Comparison of CAM and CM users.  Bivariate statistics | Interviewed about their health problems and use of healthcare services – no specification of the questions – | Female, younger, married, highly educated, spirituality important, higher occupation, higher income.  Health beliefs:  some pragmatic, some holistic ( for more spiritual CAM),  desperation with CM for more conventional CAM. Greater self reliance, belief in ability of self to help problem, internal locus of control. | CAM practitioners in the area. Recommendation by others, prior positive experience, referral. Convenience of access main reason for choosing practitioner. | | Variety of ailments, mainly chronic – more likely to perceive health problem as serious, longer term of sickness, more frequent visits to CAM, perceived intrusion of health problem of daily life. | | No use of tests of difference to establish the extent to which groups differ and whether significant. No validated measures…  5 specific CAM only | **Andersen’s model applicable to use of CAM and CM – but differences between patients who chose each modality (especially belief) even within CAM** | |
| Tsao et al [66]  2005  USA | Nationally representative probability sampling in persons receiving care for HIV.  2864 completed baseline interview (71% response rate), 2466 completed at T1 (86% response rate)  Adults 18+ yrs | | Study based on larger study HCSUS.  Prospective study, longitudinal - with two time points with interval average of 225 days.  Multivariate logistic regression. | -Predisposing: Sociodemographics, mode of HIV exposure  - Enabling: income, health insurance  - Need: HIV disease stage, wasting syndrome, CD4 count, early access to anti-retroviral therapy, pain, change in pain, vitality (  using subscales of SF36  Also: anxiety, depression, drug dependence and heavy drinking  - Outcome: CAM for HIV (differentiation between different types of CAM) | Age. |  | | Pain (greater), higher vitality, depression | | Pain measure only 2 items and for previous 4 weeks.  Not clear if all CAM therapies included. Not clear what drug use. No information about CM pain treatment - how they may have influence change in pain over time.  Only included HIV patients. | **Overall, pain as a ‘need’ characteristic of SBM predicted CAM use (few predisposing and enabling variables did). Limited support for the model.** | |
| Fouladbakhsh & Stommel [68]  2008  USA | N= 31044 participants – (89.6% response rate): comparison of cancer (n=2,262) + non cancer (n=28,734).  Sample based on national sampling strategy – highly inclusive and representative  Adults 18+ yrs | | Based on data from 2002 National Health Interview Survey, by household interview.  Multinomial logit regression. | - Predisposing: age, gender, marital status, race  - Enabling: Income, health insurance and conventional meds use,  - Need: reports of pain, symptoms, co morbid condition, health status and past diagnosis of cancer (yes/no)  - Outcome: CAM usage (provider/product/ practice-based) | Female, age (midlife 30-60), higher education and race | Income related to type of use (practitioner based vs. product based), having health insurance, use of conventional medicine. | | Co morbid conditions, having pain (especially practitioner use), greater symptoms (except product use). In cancer patients usage is reduced when greater pain, whereas in controls usage is increased with pain | | No health beliefs.  Focus on cancer | **Some differences in pattern of use between type of CAM**  **SBM all factors predicted CAM use in both populations.** | |
| Yussman et al [28]  2004  USA | N=7371  (Parents of) Children < 21 yrs | | Part of large scale, nationally representative MEPS survey.  Cross sectional. Random. Telephone/mail survey and interview.  Logistic regression | - Predisposing: age, gender, ethnicity, number of parents, education, marital status, age, region of country  - Enabling: income, poverty status.  - Need: perceived physical and mental health, number of school days/workdays missed, and physical limitation.  - Healthcare experience added: emergency department visits, inpatient discharges, medications, conventional practitioner visits, parental CAM use, immunisation status  - Outcome: practitioner based CAM use | Older age, female gender. | None | | Very good and good perceived routine health.  Also Healthcare experience: parental use of CAM (strongest predictor). | | Practitioner based CAM use only. | **Limited support of SBM (enabling not associated), but supportive of addition of healthcare experience.** | |
| **Socio behavioural model AND Health locus of control** | | | | | | | | | | | |  |
| Sirois & Gick  2002 [57]  Canada | N = 199 (155 females / 43 males, 1 transsexual)  recruited from 13 conventional medicine centres  Aim to explore differences in health beliefs and motivations between 3 groups  Adults 19+ | | Cross sectional- Self-selection  Comparison of 3 groups  Multivariate analysis | All validated and reliable measures.  - The multidimensional health locus of control scale (MHLC) form A  - Doctor satisfaction questionnaire  - Health aware behaviours questionnaire  - The negative emotionality scale (Tellegan A., Brief manual for the Differential Personality Questionnaire, unpublished)  - Five factor model of personality  - Reasons for using CAM  - Use of CAM  - Medical history  -Demographics | Health Beliefs, socio-demographics, medical and personality variables.  Independent predictors: health aware behaviours, dissatisfaction w medical doctors, number of health problems and income (representing the predisposing and enabling factors in the SBM)  Health locus of control did not predict diff between 3 groups. | | | | Study was relatively small – did not provide comparison between CAM users and Conventional med users. | | **Supportive of the utility of SBM**  **Not supportive of health locus of control.**  **Supportive of personality dimension ‘Openness’** |  |
| **Health locus of control** | | | | | | | | | | | | |
| Sasagawa et al [58]  2008  USA | N=123 – not necessarily patients- just members of the public  Adults 18+ yrs | | Cross sectional  Web-based online survey:  Not Random- invitational email  Correlational analysis | - The multidimensional health locus of control scale (MHLC form B including the internal, chance and powerful other subscales.  - Use of CAM  - Belief of the effects of CAM on health  - General health status | Females  Chronic conditions.  Internal HLOC (not conventional med use (Rho =0.26))  Frequent use of CAM associated high HLOC.  Use of CAM not confined to high HLOC, but less use of conventional med in high HLOC. | | | | Not random  Selection bias | | **HLOC correlated with CAM use, but not CM – supportive of HLC, but only through bivariate stats** | |
| Testerman, et al [59]  2004  USA | Family practice outpatients  N= 241 (42.4% response rate – similar across groups though)  Adults 40-60 yrs | | Postal survey. Comparison of patients with osteoarthritis, depression, or both were compared to healthy.  Correlational and multivariate logistic regression. | Validated, multi-item measures:  - Multidimensional Health Locus of Control Scale - Series of questions re= on holistic beliefs and spirituality – validated  - Physician care satisfaction  - Questionnaires assessing functional status  - CAM use | Health locus of control and satisfaction w care – did NOT correlate with CAM use. CAM use correlated w holistic beliefs and functional status which explained 15.8% variance in CAM use. – higher spirituality and more pain.  Correlation of CAM use w holistic beliefs, transformational experiences. | | | |  | | **No relationship to Locus of Control or care dissatisfaction. - not predictive of CAM use**  **– but a relationship to dissatisfaction w treatment.** | |
| Henderson & Donatelle [60]  2003  USA | N= 551 women w breast cancer – approached through the CM centre – 78% response rate  Adults (women) | | Computer assisted telephone interview.  Comparison of four groups – No CAM; 1+physical CAM / 1+ psychological CAM / Both physical +psychological CAM  Multinomial logistic regression | - Demographics  - Cancer Locus of control Scale. Validity and reliability established  - Outcome: type of CAM use (Differentiation between different types of CAM) | Type of CAM use related to specific perceptions of control. All types of CAM (most for users of both physical and psychological CAM) predicted by:  Perception of control over the course of the cancer. Demographics explain 23% of variance in CAM use. | | | | Low variability-as 93% had medium-high perception of Control.  No info on race, stage of disease or cancer treatment,  Cross sectional so no causality | | **Locus of control useful as predictor of cam use** | |
| Hedderson et al [61]  2004  USA | Cancer patients  N=356 (response rates 32% to 35%). 50% female.  Adults 20 – 79 yrs | | Cross sectional telephone survey. Random sample from Cancer Surveillance System.  Logistic regression | - Demographics  - Adapted Desire for Dental Control scale  - Wallston MHLoC-Internal health locus of control scale  - Quality of Life  - Self rated health status  - Outcome: CAM practitioner and products, last 12 months.  Logistic regression | Female  High symptom distress  Desire for personal control for supplements only. | | | | Cancer only. Lack of support for Internal HLoC may be due to cancer subset (measure not specific to them). Sample size underpowered. | | **Internal HLoC not predictive.** | |
| **Theory of Planned Behaviour AND Transtheoretical model AND Self Efficacy** | | | | | | | | | | | | |
| Hirai et al [62]  2008  Japan | N = 521  246 males and 270 females from three cancer treatment centres (response rate 59.2% - rate of valid replies 47.4)  Age range not given | | Multi-centre cross sectional survey – Convenience sample  Univariate and multivariate analysis of variance. | CAM Use, patient background: disease, age at onset, age, gender, education, income, type of cancer treatment, satisfaction with treatment, smoking, drinking, social support  TPB and TTM related questions  HADS, Self efficacy (Perceived control)  MD Anderson Symptom Inventory (JDASI-J)  Analysis included structural equation modelling and differences in group means (ANOVA) | 41 % variance related more advance stages of CAM use through the TTM and TPB variables were: expectation from family members, pros, norms of medical staff and cons.  Other factors associated with higher stages of CAM use in TTM: Patients receiving chemotherapy (p<0.001), patients dissatisfied with current conventional treatment (p<0.05) and outpatients (p< 0.001) | | | | Low response rate. Convenience sample | | **Psychological factors more important than medical and demographic variables.**  **Supports use of TTM and TPB together.** | |
| **Treatment beliefs and illness beliefs (within the self-regulatory model)** | | | | | | | | | | | | |
| Bishop & Yardley [69]  2006  UK | n=247 Participants recruited through health-related internet websites and bulletin boards. Response rate %64. Considered themselves to have health problem.  (92% female).  Adults 20-60 yrs | | Correlational web based questionnaire (for 6 months)  Multiple logistic regression. | Validated measures – all multi item reliable  Treatment beliefs * CAM beliefs inventory (Validated and reliable_ - treatment beliefs)  *Beliefs Medicine Questionnaire - beliefs about use of meds  - Attitudes to GPs  - Revised Illness Perceptions Questionnaire – validated/reliable  -Illness beliefs  - Demographics | Holistic beliefs (particularly: strong understanding of illness, strong belief that illness and serious consequences, and that emotional factors caused illness).  Majority of illness beliefs and treatment beliefs not associated with CAM use (and demographics explained variance) | | | | Limited sampling (self-selected computer literate internet users) : mostly female, young, educated – which would limit range.  Self report of illness.  . | | **Only a minority of illness and treatment beliefs predictive of CAM use – therefore limited support for self-regulation model** | |
| **Braden’s Self help model** | | | | | | | | | | | | |
| Owens et al [71]  2007  USA | N=140 . Hispanic women. Convenience sample of women receiving treatment for breast cancer in outpatient clinic.  Adults | | Descriptive, correlational- cross sectional  Multiple logistic regression. | 9 Scales- all verified to have high internal consistency & reliability. | CAM use was not shown to be a resource to reduce uncertainty in this population. CAM use shown to be correlated w perception of CAM efficacy and family income | | | | - no info re cancer treatments  - Sources of error introduced in the process of filling out questionnaires as  - no differentiation between types of CAM –may be underreported | | **Strong support for Braden’s self help model.** | |
| **Consumer decision-making model** | | | | | | | | | | | | |
| Sirois & Purc-Stephenson [43]  2008  Canada | Recruited from both CM and CAM institutions.  Questionnaires distributed in 27 health clinics – N=239 in total (35.6% response rate- nearly 59% in CM setting).  Adults 15 and over | | Quantitative cross-sectional  Comparison of 3 groups – CM, new or infrequent CAM and Established CAM.  Multinomial logistic regression. | Scales mostly used previously or validated.  Demographics, health problems, use of CAM, satisfaction w CM doctors, beliefs about healthcare practitioner’s role, attitudes towards CAM and reason for using CAM  -Patient satisfaction questionnaire – validated  - A modified Beliefs about healthcare professionals scale (alpha =.75)-reliable | Predictors of new/infrequent/established CAM use:  greater symptom distress, stronger expectation for egalitarian practitioner (decision process factors) and less satisfaction with technical aspects of CM (Postdecision factors). In both groups the decision to try CAM was motivated by other’s recommendations (external influence) and ineffective CM treatment (postdecision) | | | | Not specifically related to health  representative-ness  did not incorporate a full array of decision _making factors relating to emotional and interactive aspects of care | | **Good model for understanding role of CAM use throughout the decision process** | |
| **Ethnographic Decision Tree Modelling (developed and tested their own model)** | | | | | | | | | | | | |
| Montbriand [70]  1995  Canada | | Cancer patients.  5 hospitals in Prairie. N=48 for tree development, 252 for testing.  Randomly assigned.  Adults 35 – 80 yrs | Gladwin’s methodology.  Interviews to develop a decision tree which is then tested with a separate sample. | Open ended questions based on the decision tree.  New model but tested. Very good predictability. Member checks and triangulation re qualitative data. | Main themes:  - Social group influence  - Cost consideration  - Stress  - Desired control  - Judgements about change, cure and searching  - Faith in practice  Predictability of model = 90.4%  56 were high interest (in CAM), 121 were regular (both), 75 were biomedical only | | | | Cancer only | | **Developed a new model with very good predictability.** | |
| **Decision making process – not defined** | | | | | | | | | | | | |
| Caspi, et al [76]  2004  USA | N = 12 (58% female) (All groups suffer chronic rheumatologic disorders. Purposive sampling  from CM (75%) and community CAM practices.  Adults (age range not given) | | Naturalistic – using Qualitative methods (thematic and content analysis)  Exploratory/cross-sectional | In-depth open ended interviews Comparison of 3 groups: CM (who considered CAM but didn’t use in the end), CAM only and mixed CAM/CM: | Groups differed in :  Trust in their physicians, weight placed on scientific evidence, comfort with uncertainty /trust, perceptions of the influence of disease severity on seeking CAM.  - Allopathic group:  Continuous appraisal of well being and functionality- leading to consideration of alt treatment option, by deciding on 1)positive/negative consequences, 2)severity of condition, 3) prognosis. Trust in allopathic healthcare system, but willingness to try CAM if all else fails.  - Alternative group:  Decision to seek CAM based on 1) recommendations, 2) repetitive endorsement of treatment. Participants willing to experiment, less trust in allopathy, holistic approach- intuitive.  - Complementary group:  Required multiple sources of data to make decision, educated choice, continuous evaluation of relief, congruency with belief system and cost – diff approaches to. | | | | Not random (but useful in gaining maximum richness of the data) | | **Identified the differences between types of CAM users. Proposed a model but this was not tested, and needs further naturalistic development.** | |
| Truant & Bottorff [72]  1999  USA | Purposive sampling of 16 women with breast cancer | | Qualitative analysis using grounded theory.  Adults (women) 39 – 71 yrs | Open ended, exploratory unstructured interview | Decision to use CAM revisited and re-evaluated /modified over time.  3 stages to mode of process:  phase 1 : getting something in place; 2: hand picking CT that suit one, 3: Living /fine tuning choice.  The importance of CONTROL – provided a strategy to assume control over their disease, treatment and well-being | | | | Not entirely a model- but depiction of the process in this specific population | | **Models change over time.**  **Control is important.** | |
| Bishop et al 73]  2008  UK | Recruited from CAM clinic  N = 42 (96% female)  Ages not given | | Qualitative analysis | Open ended questions about choice of therapy, therapist, experience & perceptions.  Differentiation between different types of CAM. | CAM therapy seen as either a treat or a treatment. Treatments were viewed wither as complementary, alternative or conventional: this was construed according to the extent of medical need and the extent to which treatment was viewed as holistic | | | | Although explains perceptions of clients of these treatments – does not give a wider understanding of what predicts initiation or use of CAM | | **Does not expand on a model** | |
| Singh et al [75]  2005  USA | Males with prostate cancer. 27 interviewed (whole sample N=1168).  Adults 58 – 82 y | | Part of a larger study. Open ended interviews. | Open ended questions on demographics, satisfaction with healthcare practitioner, conventional treatment, CAM used, CAM practitioners, communication, costs of treatment, effectiveness of treatments, dietary patterns, physical activity, religion, perceptions about causes of cancer, goals of treatment and sources of information. | Four major themes:  - Perceived goals of CAM and conventional medicine  - Concerns about side effects  - Belief in efficacy of CAM  - Sense of control | | | | Model they developed not tested (qualitative only).  Prostate cancer only. | | **Interactive nature of factors in a model is important.** | |
| Balneaves et al [74]  2007  Canada | N=20  Women with breast cancer. Purposive sampling from clinical oncology settings.  Adults 37 to 59 yrs | | Qualitative, grounded theory. | In depth semi structured interviews on personal, social and system factors that influence treatment choices, information sources and challenges making decisions. | Patients viewed a ‘gap’ between CAM and CM in paradigm, information and role. 3 ways to bridge the gap:  - One step at a time: slow down CAM use, increased information seeking  - Playing it safe: trusting CM, using little CAM  - Bringing it all together: previous CAM users, use both for wellbeing.  Groups are dynamic and patients move between categories | | | | Model not tested. Breast cancer only. Didn’t include non users of CAM. | | **Developed their own model: “Bridging the gap”** | |
| **CM = Conventional medicine**  **CAM = complementary and alternative medicine** | | | | | | | | | | | | |
